# Nonstationary Model of Oxygen Transport in Brain Tissue

**DOI:** 10.1155/2020/4861654

**Published:** 2020-07-11

**Authors:** Andrey E. Kovtanyuk, Alexander Yu. Chebotarev, Nikolai D. Botkin, Varvara L. Turova, Irina N. Sidorenko, Renée Lampe

**Affiliations:** ^1^Klinikum rechts der Isar, Technische Universität München, Ismaningerstr. 22, 81675 München, Germany; ^2^Far Eastern Federal University, Sukhanova st. 8, 690950 Vladivostok, Russia; ^3^Institute for Applied Mathematics FEB RAS, Radio st. 7, 690041 Vladivostok, Russia; ^4^Fakultät für Mathematik, Technische Universität München, Boltzmannstr. 3, 85747 Garching bei München, Germany

## Abstract

The paper addresses the mathematical study of a nonstationary continuum model describing oxygen propagation in cerebral substance. The model allows to estimate the rate of oxygen saturation and stabilization of oxygen concentration in relatively large parts of cerebral tissue. A theoretical and numerical analysis of the model is performed. The unique solvability of the underlying initial-boundary value problem for a system of coupled nonlinear parabolic equations is proved. In the numerical experiment, the tissue oxygen saturation after hypoxia is analyzed for the case when a sufficient amount of oxygen begins to flow into the capillary network. A fast stabilization of the tissue oxygen concentration is demonstrated. The reliability of the results of the numerical simulation is discussed.

## 1. Introduction

The main requirement of proper functioning of the brain is its sufficient supply with oxygen. Scarcity of oxygen caused, for example, by the decrease of blood circulation can provoke injuring and death of brain cells. Therefore, it is necessary to better recognize all factors affecting oxygen transport in the brain. In this connection, mathematical modeling can be very helpful to understand the cause of impaired oxygen delivery.

There are a great number of mathematical models of oxygen transport in the brain. Among them, the method, where the cerebral substance is regarded as a bifractional (blood and tissue) homogenized material, becomes more and more popular. Mathematically speaking, such models are represented by coupled partial differential equations describing convection, diffusion, and consumption of oxygen in blood and tissue fractions (see, e.g., [1–9]).

As a rule, the concentrations of oxygen in the blood and tissue fractions are distributed state variables of the homogenized models. However, such models should reflect the processes occurring in the prehomogenization phase such as oxygen penetration from blood plasma to tissue through capillary walls, which requires taking into account the plasma oxygen concentration. The nonlinear Hill equation describes a dependence between the oxygen concentrations in plasma and blood, so that the new state variable does not appear in the coupling between the blood and tissue compartments of the resulting homogenized model. It should also be mentioned the presence of a sink term in the tissue-fraction model equation, which describes oxygen consumption in tissue. This term is derived using the Michaelis-Menten equation. Therefore, the above outlined two-compartment models consisting of two coupled quasilinear parabolic equations are difficult for mathematical analysis and numerical implementation. This is the reason why many researchers have tried to simplify the models. Below, an outline of some investigations addressing various models of oxygen transport in the brain is presented.

Mathematical steady-state models of oxygen propagation in a tissue comprising a capillary network are presented in papers [1–4]. The method used there is based on Green's function techniques. In [1], some assumptions such as the neglect of blood plasma oxygen and the independence of metabolic rate on tissue oxygen concentration are imposed. This allows for reducing the degree of nonlinearity in the model. In [2–4], using modifications of the method proposed in [1], the authors consider the case of nonuniform consumption. Moreover, in [4], free oxygen dissolved in blood plasma is taken into account.

Nonstationary models of oxygen transport are the subject of research in many works (see, e.g., [5–11]). Using time-dependent models, it is possible to simulate transition regimes of oxygen transfer such as the saturation of tissues with oxygen.

Paper [5] presents a one-dimensional oxygen transport model containing both nonlinear differential and algebraic equations describing the oxygen concentrations in blood, plasma, and tissue. It should be stressed that the model spatially averages the concentration of oxygen in the tissue fraction.

In [6], a hybrid oxygen transport model is considered. It comprises a one-dimensional equation describing the oxygen transfer in the vessel, a one-dimensional conservation equation for oxygen flux through blood-tissue interface, and a three-dimensional diffusion equation, with a consumption term, governing oxygen propagation in tissue. Thus, there are three computational domains, each for its corresponding equation. The method proposed yields consistent results.

Paper [7] proposes a model of oxygen transportation to living cells. The consideration is done at the scale that allows for taking individual red blood cell into account. The model predicts partial oxygen pressure in capillaries and neighboring tissue areas.

In [8], oxygen transfer from blood to tissue is modeled using a two-compartment model operating with blood and tissue fractions. Numerical tests were conducted for relatively large vessel networks. The tissue oxygenation accounting for hematocrit distribution is computed.

In [9], on the base of a new model of the cerebral microvasculature, a nonstationary model of oxygen transport is considered. The vascular and tissue responses to changes in flow and metabolism are studied. Concerning the main assumptions of this approach, the spatial structure of the network is not taken into account, the diffusion within the tissue is neglected, and the metabolic rate is supposed to be independent on tissue oxygen concentration. The advantage of the proposed approach is its ability to be applied to a large enough volume of tissue.

A perspective trend in modeling oxygen transport is related to the so-called continuum models obtained through spatial homogenization of state variables. In [12], such a method is used to simulate heat processes in a tissue comprising a network of blood vessels. In the resulting model, the same spatial domain stands for blood and tissue fractions. A similar ansatz is used in [10, 11, 13, 14], where continuum models governed by coupled partial differential equations are studied. In [10, 11], numerical simulations of a nonstationary continuum model were performed in case of simple domains, and the comparison of results with those obtained for the original (nonhomogenized) model is done. In [13], a theoretical analysis of steady-state oxygen transport model is fulfilled. A priori estimates of solutions, implying the unique solvability of the problem under some conditions, are obtained. An iterative numerical procedure for finding solutions is proposed, along with the proof of its convergence. In [14], the investigation of steady-state continuum models is continued, the existence and uniqueness of solutions for the boundary-value problem are established, and numerical examples that illustrate the theoretical analysis are computed.

Despite the intensive numerical simulations of continuum models of oxygen transport, mathematical analysis of underlying partial differential equations is seldom addressed. The exception is a theoretical analysis of steady-state models considered in [13, 14]. As for nonstationary models, theoretical issues related to the existence and uniqueness of solutions for underlying initial-boundary value problems are still open. The aim of the present paper is to perform accurate mathematical analysis of the underlying nonstationary nonlinear initial-boundary value problem, establish its unique solvability, and implement an iterative solution algorithm based on Finite Element Method. It should be noted that the results of numerical experiments demonstrate a fast stabilization of the oxygen concentration due to diffusion and consumption effects.

Notice that homogenization leads to averaging of oxygen concentrations and smoothing the gradients. In particular, in contrast to the approaches considered in [1–4, 6–8], homogenization of vascular networks does not allow for observing gradients around single capillaries. Nevertheless, this approach allows to simulate other important processes such as diffusion, convection, and consumption of oxygen in relatively large parts of cerebral tissue. Moreover, as demonstrated in numerical experiments, it is possible to estimate the rate of tissue oxygen saturation and the corresponding stabilization of oxygen propagation on the base of the continuum model studied. Additionally, the approach proposed makes it possible to carry out a theoretical analysis of underlying initial-boundary value problems using their weak formulations. The use of continuum models enables to take into account most of the important effects of oxygen transport, for example, the dependence of the metabolic rate on the tissue oxygen concentration, amount of free oxygen dissolved in blood plasma, and diffusion rate of oxygen, which, however, was not always considered in the above cited works.

## 2. Problem Formulation

We consider the vessel-tissue system as a two-phase flow system, including the blood phase with the volume fraction *σ* and the tissue phase with volume fraction 1 − *σ*.

Similar to other two-phase flow models, the oxygen concentrations in the blood and tissue phases are governed by the following coupled parabolic equations (cf. [5, 10, 11]):

(1) $$\begin{array}{c} \frac{\partial \varphi }{\partial t}-\alpha \Delta \varphi +v\cdot \nabla \varphi =G,\\ \frac{\partial \theta }{\partial t}-\beta \Delta \theta =-\kappa G-\mu ,\;x\in \Omega ,\;t\in \left(0,T\right). \end{array}$$

Here, *φ* and *θ* are the blood and tissue oxygen concentrations, respectively, *μ* describes the tissue oxygen consumption, *G* is the intensity of oxygen exchange between the blood and tissue fractions, *κ* = *σ*(1 − *σ*)^−1^, *σ* is the volumetric fraction of vessels, **v** is a prescribed continuous velocity vector field in the entire domain *G* (the averaging of the velocity field of the capillary network), and *α* and *β* are diffusivity parameters of the corresponding phases. Notice that, as a result of homogenization, the blood and tissue oxygen concentrations are defined in the same continuum domain *Ω*.

The Michaelis-Menten equation describes the tissue oxygen consumption rate, *μ*, as function of *θ*, the oxygen concentration in tissue, as follows:

(2) $$\begin{array}{c} \mu =\mu \left(\theta \right)≔\frac{\mu_{0}\theta }{\theta +\theta_{50}}, \end{array}$$

where *μ*_0_ is the maximum value of *μ* and *θ*_50_ is the value of *θ* at *μ* = 0.5*μ*_0_.

The transfer rate of oxygen from blood to tissue through vessel walls is given by the formula

(3) $$\begin{array}{c} G=a\left(\theta -\psi \right),\\ \varphi =f\left(\psi \right)≔\psi +\frac{b\psi^{r}}{\psi^{r}+c}, \end{array}$$

where *ψ* is the oxygen concentration in plasma (concentration at vessel walls). Note that *ψ* is expressed through *φ* so that *ψ* is not a state variable of the resulting model. Additionally, positive constants *a*, *b*, *c*, and *r* have the following sense: *a* defines the oxygen permeability, *b* characterizes the concentration of tetramer hemoglobin, and *c* is given by the formula *c* = *ψ*_*H*_^*r*^, where *ψ*_*H*_ is the concentration of oxygen in plasma at the hemoglobin level of 50%, and *r* is the Hill exponent (or coefficient).

We assume that the oxygen concentrations *φ* and *θ* satisfy the following conditions on the boundary Γ = *∂Ω*:

(4) $$\begin{array}{c} {\left.\alpha \partial_{n}\varphi +\gamma \left(\varphi -\varphi_{b}\right)\right|}_{\Gamma }=0,\\ \beta \partial_{n}\theta +\delta \left(\theta -g\left(\varphi_{b}\right)\right)\left|{}_{\Gamma }=0,\right. \end{array}$$

and the following initial conditions:

(5) $$\begin{array}{c} \varphi \left|{}_{t=0}=\varphi_{0}\right.,\\ \theta \left|{}_{t=0}=\theta_{0}\right.. \end{array}$$

Here, *∂*_*n*_ is the outward normal derivative at points of the domain boundary. Nonnegative functions *φ*_*b*_ = *φ*_*b*_(*x*), *γ* = *γ*(*x*), *δ* = *δ*(*x*), *x* ∈ Γ, and the initial functions *φ*_0_ = *φ*_0_(*x*) and *θ*_0_ = *θ*_0_(*x*), *x* ∈ *Ω*, are given. The function *g* is defined as the inverse of *f*.

## 3. Problem Formalization and a Priori Estimates

Let *Ω* be a bounded Lipschitz domain with the boundary Γ = *∂Ω* consisting of finite number of smooth parts. Set *Q* = *Ω* × (0, *T*) and *Σ* = Γ × (0, *T*). Denote by *L*^*p*^, 1 ≤ *p* ≤ ∞, the space of *p*-integrable (essentially bounded if *p* = ∞) functions. Let *H*^*s*^ be the Sobolev space *W*_2_^*s*^. The space *L*^*s*^(0, *T*; *X*) (respectively, *C*([0, *T*]; *X*)) consists of *s*-integrable on (0, *T*) (respectively, continuous on [0, *T*]) functions assuming values in a Banach space *X*.

Suppose that the following conditions hold for the model data:

(6) $$\begin{array}{c} \gamma ,\delta \in L^{\infty }\left(\Gamma \right),\;\gamma \geq \gamma_{0}>0,\;\delta \geq \delta_{0}>0,\\ \varphi_{b}\in L^{2}\left(\Sigma \right),\\ \theta_{0},\varphi_{0}\in L^{2}\left(\Omega \right),\\ v\in L^{\infty }\left(Q\right), \end{array}$$

where *γ*_0_ and *δ*_0_ are constants.

Denote *H* = *L*^2^(*Ω*), *V* = *H*^1^(*Ω*), and *V*′ the dual of *V*. Identify *H* with its dual *H*′ to get the Gelfand triple *V* ⊂ *H* = *H*′ ⊂ *V*′. Let ‖·‖, ‖·‖_*V*_, and ‖·‖_∗_ denote the norms in *H*, *V*, and *V*′, respectively. Notice that (*f*, *v*) is the value of functional *f* ∈ *V*′ on an element *v* ∈ *V*. If *f* ∈ *H*, then (*f*, *v*) is the inner product in *H*.

Introduce the inner product in *V* by the relation

(7) $$\begin{array}{c} \left(\left(f,g\right)\right)=\left(f,g\right)+\left(\nabla f,\nabla g\right). \end{array}$$

Define the following space:

(8) $$\begin{array}{c} W=\left\{y\in L^{2}\left(0,T;V\right)\text{: }y^{\prime }\in L^{2}\left(0,T;V^{\prime }\right)\right\}, \end{array}$$

where *y*′ = *dy*/*dt*. It is well known that *W* ⊂ *C*([0, *T*]; *H*) is the continuous embedding.

Remembering the problem formulation, introduce strictly increasing odd functions *μ* : ℝ⟶ℝ and *f* : ℝ⟶ℝ defined by the formulas

(9) $$\begin{array}{c} \mu \left(\lambda \right)≔\frac{\mu_{0}\lambda }{\lambda +\theta_{50}},\\ f\left(\lambda \right)≔\lambda +\frac{b\lambda^{r}}{\lambda^{r}+c},\;\lambda \geq 0. \end{array}$$

Let *g* : ℝ⟶ℝ denote the inverse of *f*. Note that

(10) $$\begin{array}{c} \left|\mu \left(\lambda \right)\right|\leq \mu_{0},\\ \left|g\left(\lambda \right)\right|\leq \left|\lambda \right|,\\ 0\leq \mu^{\prime }\left(\lambda \right)\leq \frac{\mu_{0}}{\theta_{50}},\\ 0\leq g^{\prime }\left(\lambda \right)\leq 1,\;\lambda \in \mathbb{R}. \end{array}$$

Define operators *A*_1,2_ : *V*⟶*V*′ and functionals *f*_1,2_ ∈ *L*^2^(0, *T*; *V*′) using the following relations:

(11) $$\begin{array}{c} \left(A_{1}u,v\right)=\alpha \left(\nabla u,\nabla v\right)+\int_{\Gamma }\gamma uvd\Gamma ,\\ \left(A_{2}w,v\right)=\beta \left(\nabla w,\nabla v\right)+\int_{\Gamma }\delta wvd\Gamma ,\\ \left(f_{1},v\right)=\int_{\Gamma }{\gamma \varphi }_{b}vd\Gamma ,\\ \left(f_{2},v\right)=\int_{\Gamma }\delta g\left(\varphi_{b}\right)vd\Gamma \text{ a}.\text{e}.\text{ on }\left(0,T\right), \end{array}$$

where *u*, *w*, *v* ∈ *V* are arbitrary functions. Notice that the bilinear forms (*A*_1_*y*, *z*) and (*A*_2_*y*, *z*) define inner products in *V*, and the following inequalities hold:

(12) $$\begin{array}{c} \left(A_{1}y,y\right)\geq k_{1}{\left\|y\right\|}_{V}^{2},\\ \left(A_{2}y,y\right)\geq k_{2}{\left\|y\right\|}_{V}^{2}, \end{array}$$

where positive constants *k*_1_, *k*_2_ do not depend on *y* ∈ *V*.

Therefore, the problem (1)–(5) can be rewritten as a Cauchy problem in an operator form.

**Definition 1 .**

A pair {*φ*, *θ*} ∈ *W* × *W* is a weak solution of the problem (1)–(5) if

(13) $$\begin{array}{c} \varphi^{\prime }+A_{1}\varphi +v\nabla \varphi +a\left(g\left(\varphi \right)-\theta \right)=f_{1}\text{ a}.\text{e}.\text{ on }\left(0,T\right), \end{array}$$

(14) $$\begin{array}{c} \theta^{\prime }+A_{2}\theta +\mu \left(\theta \right)+\kappa a\left(\theta -g\left(\varphi \right)\right)=f_{2}\text{ a}.\text{e}.\text{ on }\left(0,T\right), \end{array}$$

(15) $$\begin{array}{c} \varphi \left|{}_{t=0}=\varphi_{0}\right., \end{array}$$

(16) $$\begin{array}{c} \theta \left|{}_{t=0}=\theta_{0}\right.. \end{array}$$

**Remark 1 .**

The above definition can be rewritten in an operator form as follows. A pair *u* = {*φ*, *θ*} is a weak solution iff it solves the equation *u*′ + *Lu* = *f*, where *f* = {*f*_1_, *f*_2_}, and *L* is the operator defined by the left-hand sides of (13) and (14). Unfortunately, as it is shown in [14], the operator *L* is not monotone for typical values of problem parameters, which prevents from applying standard methods of analysis (cf. [15]).

## 4. The Existence and Uniqueness of Solution

**Theorem 1 .**

Let conditions (6) hold. Then, the problem (1)–(5) is unique solvable on any finite time interval [0, *T*], 0 < *T* < ∞.

**Proof**

Define Galerkin approximations *φ*_*m*_ and *θ*_*m*_ of solutions of the problem (1)–(5) and derive a priori estimates which are necessary to prove the solvability. To do that, introduce a basis *w*_1_, *w*_2_, ⋯ of *V* such that these functions are orthonormal in *H*. Let

(17) $$\begin{array}{c} \varphi_{m}\left(t\right),\theta_{m}\left(t\right)\in V_{m}=\text{span}\left\{w_{1},\cdots ,w_{m}\right\},\;t\in \left(0,T\right), \end{array}$$

satisfy the relations

(18) $$\begin{array}{c} \begin{array}{cc} \left(\varphi_{m}^{\prime }+A_{1}\varphi_{m}+v\nabla \varphi_{m}+a\left(g\left(\varphi_{m}\right)-\theta_{m}\right)-f_{1},v\right)=0, & \\ \forall v\in V_{m};\;\varphi_{m}\left(0\right)=\varphi_{0m}, \end{array} \end{array}$$

(19) $$\begin{array}{c} \left(\theta_{m}^{\prime }+A_{2}\theta_{m}+\mu \left(\theta_{m}\right)+\kappa a\left(\theta_{m}-g\left(\varphi_{m}\right)\right)-f_{2},w\right)=0, \end{array}$$

where *φ*_0*m*_ and *θ*_0*m*_ are *H*-orthonormal projections of the functions *φ*_0_ and *θ*_0_ on the subspace *V*_*m*_.

Assuming *v* = *φ*_*m*_ and *w* = *θ*_*m*_ in (18) and (19), adding these equations, taking into account properties (10) and (12) as well as the nonnegativity of the products *g*(*φ*)*φ* and *μ*(*θ*)*θ*, yield the following inequality:

(20) $$\begin{array}{c} \frac{1}{2}\frac{d}{dt}\left({\left\|\varphi_{m}\right\|}^{2}+{\left\|\theta_{m}\right\|}^{2}\right)+k_{1}{\left\|\varphi_{m}\right\|}_{V}^{2}+k_{2}{\left\|\theta_{m}\right\|}_{V}^{2}\leq \\ \leq {\left\|f_{1}\right\|}_{V^{\prime }}{\left\|\varphi_{m}\right\|}_{V}+{\left\|f_{2}\right\|}_{V^{\prime }}{\left\|\theta_{m}\right\|}_{V}+{\left\|v\right\|}_{L^{\infty }\left(Q\right)}\left\|\nabla \varphi_{m}\right\|\left\|\varphi_{m}\right\|+a\left(\kappa +1\right)\left\|\theta_{m}\right\|\left\|\varphi_{m}\right\|. \end{array}$$

The terms of the last inequality are being estimated using the relation *uv* ≤ *εu*^2^/2 + *v*^2^/2*ε* holding for all *ε* > 0. Taking *ε* = *k*_1_/2, *ε* = *k*_2_, and *ε* = 1 yields

(21) $$\begin{array}{c} {\left\|f_{1}\right\|}_{V^{\prime }}{\left\|\varphi_{m}\right\|}_{V}\leq \frac{k_{1}}{4}{\left\|\varphi_{m}\right\|}_{V}^{2}+\frac{1}{k_{1}}{\left\|f_{1}\right\|}_{V\prime }^{2},\\ {\left\|f_{2}\right\|}_{V^{\prime }}{\left\|\theta_{m}\right\|}_{V}\leq \frac{k_{2}}{2}{\left\|\theta_{m}\right\|}_{V}^{2}+\frac{1}{2k_{2}}{\left\|f_{2}\right\|}_{V\prime }^{2},\\ {\left\|v\right\|}_{L^{\infty }\left(Q\right)}\left\|\nabla \varphi_{m}\right\|\left\|\varphi_{m}\right\|\leq \frac{k_{1}}{4}{\left\|\varphi_{m}\right\|}_{V}^{2}+\frac{1}{k_{1}}{\left\|v\right\|}_{L^{\infty }\left(Q\right)}^{2}{\left\|\varphi_{m}\right\|}^{2},\\ a\left(\kappa +1\right)\left\|\theta_{m}\right\|\left\|\varphi_{m}\right\|\leq a\left(\kappa +1\right)\frac{\left({\left\|\theta_{m}\right\|}^{2}+{\left\|\varphi_{m}\right\|}^{2}\right)}{2}. \end{array}$$

Along with the integration over *t*, this yields the following estimate:

(22) $$\begin{array}{c} \begin{array}{c} {\left\|\varphi_{m}\left(t\right)\right\|}^{2}+{\left\|\theta_{m}\left(t\right)\right\|}^{2}+\int_{0}^{t}\left(k_{1}{\left\|\varphi_{m}\left(s\right)\right\|}_{V}^{2}+k_{2}{\left\|\theta_{m}\left(s\right)\right\|}_{V}^{2}\right)ds\leq \\ \leq C_{1}+C_{2}\int_{0}^{t}\left({\left\|\varphi_{m}\left(s\right)\right\|}^{2}+{\left\|\theta_{m}\left(s\right)\right\|}^{2}\right)ds. \end{array} \end{array}$$

Here,

(23) $$\begin{array}{c} C_{1}={\left\|\varphi_{0}\right\|}^{2}+{\left\|\theta_{0}\right\|}^{2}+\int_{0}^{T}\left(\frac{2}{k_{1}}{\left\|{ƒ}_{1}\left(s\right)\right\|}_{V\prime }^{2}+\frac{1}{k_{2}}{\left\|{ƒ}_{2}\left(s\right)\right\|}_{V\prime }^{2}\right)ds,\\ C_{2}=\frac{2}{k_{1}}{\left\|v\right\|}_{L^{\infty }\left(Q\right)}^{2}+a\left(1+\kappa \right). \end{array}$$

Applying the Gronwall inequality implies the claim

(24) $$\begin{array}{c} \varphi_{m},\theta_{m}\text{ are bounded in }L^{\infty }\left(0,T;H\right)\text{ and }L^{2}\left(0,T;V\right). \end{array}$$

Obtain now an estimate guaranteeing the compactness of the sequences *φ*_*m*_, *θ*_*m*_ in *L*^2^(0, *T*; *H*). For this end, set *v* = *φ*_*m*_(*t*) − *φ*_*m*_(*s*) in (18), integrate the result over *t* ∈ (*s*, *s* + *h*), and integrate that over *s* ∈ (0, *T* − *h*), where *h* > 0 is sufficiently small. Finally,

(25) $$\begin{array}{c} \frac{1}{2}\int_{0}^{T-h}{\left\|\varphi_{m}\left(s+h\right)-\varphi_{m}\left(s\right)\right\|}^{2}ds=\int_{0}^{T-h}\int_{s}^{s+h}c_{m}\left(t,s\right)dtds, \end{array}$$

where

(26) $$\begin{array}{c} c_{m}\left(t,s\right)=\left(A_{1}\varphi_{m}\left(t\right)+v\left(t\right)\nabla \varphi_{m}\left(t\right)+a\left(g\left(\varphi_{m}\left(t\right)\right)-\theta_{m}\left(t\right)\right)-f_{1}\left(t\right),\varphi_{m}\left(s\right)-\varphi_{m}\left(t\right)\right). \end{array}$$

Notice that, accounting for the continuity of embedding *V* ⊂ *H*, the following estimate holds:

(27) $$\begin{array}{c} c_{m}\left(t,s\right)\leq C\left({\left\|\varphi_{m}\left(t\right)\right\|}_{V}^{2}+{\left\|\theta_{m}\left(t\right)\right\|}_{V}^{2}+{\left\|\varphi_{m}\left(s\right)\right\|}_{V}^{2}+{\left\|{ƒ}_{1}\left(t\right)\right\|}_{V^{\prime }}^{2}\right). \end{array}$$

Here and below, *C* is a positive, independent on *m* constant. The terms in (27), that depend on *t*, can be estimated by changing the integration order in (25). Along with the boundedness of *φ*_*m*_ and *θ*_*m*_ in *L*^2^(0, *T*; *V*), this yields the following equicontinuity estimate:

(28) $$\begin{array}{c} \int_{0}^{T-h}{\left\|\varphi_{m}\left(s+h\right)-\varphi_{m}\left(s\right)\right\|}^{2}ds\leq Ch. \end{array}$$

Similarly, the equicontinuity of the sequence *θ*_*m*_ can be shown:

(29) $$\begin{array}{c} \int_{0}^{T-h}{\left\|\theta_{m}\left(s+h\right)-\theta_{m}\left(s\right)\right\|}^{2}ds\leq Ch. \end{array}$$

The estimates (24)–(29) allow us to pass, up to subsequences, to the limit. There are functions *φ* and *θ* such that

(30) $$\begin{array}{c} \varphi_{m}⟶\varphi ,\theta_{m}⟶\theta \text{ weakly in }L^{2}\left(0,T;V\right),\\ {}*-\text{weakly in }L^{\infty }\left(0,T;H\right),\text{ and strongly in }L^{2}\left(Q\right). \end{array}$$

Convergences listed in (30) allow us to pass to the limit in (18) and (19), as *m*⟶∞, to prove that the limiting functions *φ* and *θ* satisfy equations (13) and (14) in the sense of distributions on (0, *T*) and the initial conditions hold. Note that the passage to the limit in terms containing *µ*(*θ*_*m*_) and *g*(*φ*_*m*_) can be easily done due to the following inequalities:

(31) $$\begin{array}{c} \left|g\left(\varphi_{m}\right)-g\left(\varphi \right)\right|\leq \left|\varphi_{m}-\varphi \right|,\\ \left|\mu \left(\theta_{m}\right)-\mu \left(\theta \right)\right|\leq \frac{\mu_{0}}{\theta_{50}}\left|\theta_{m}-\theta \right|, \end{array}$$

which follows from the estimates of the derivatives of the functions *g* and *μ*. Note also that estimate (24) implies the inclusions

(32) $$\begin{array}{c} A_{1}\varphi +v\nabla \varphi +a\left(g\left(\varphi \right)-\theta \right)-f_{1}\in L^{2}\left(0,T;V^{\prime }\right),\\ A_{2}\theta +\mu \left(\theta \right)+\kappa a\left(\theta -g\left(\varphi \right)\right)-f_{2}\in L^{2}\left(0,T;V^{\prime }\right), \end{array}$$

which means that the time derivatives *φ*′ and *θ*′ belong to the space *L*^2^(0, *T*; *V*′) and satisfy equations (13) and (14) almost everywhere on (0, *T*). Thus, the pair {*φ*, *θ*} ∈ *W* × *W* is a solution of (1)–(5) in the weak sense.

Show now the uniqueness of weak solutions. Let {*φ*_1_, *θ*_1_} and {*φ*_2_, *θ*_2_} be two solutions of the problem (1)–(5), and *φ* = *φ*_1_ − *φ*_2_, *θ* = *θ*_1_ − *θ*_2_. Then, the following equalities hold:

(33) $$\begin{array}{c} \left(\varphi^{\prime }+A_{1}\varphi +v\nabla \varphi +a\left(g\left(\varphi_{1}\right)-g\left(\varphi_{2}\right)-\theta \right),v\right)=0,\;\forall v\in V;\;\varphi \left(0\right)=0,\\ \left(\theta^{\prime }+A_{2}\theta +µ\left(\theta_{1}\right)-µ\left(\theta_{2}\right)+\kappa a\left(\theta -g\left(\varphi_{1}\right)-g\left(\varphi_{2}\right)\right),w\right)=0,\;\forall w\in V;\;\theta \left(0\right)=0. \end{array}$$

Setting *v* = *φ* and *w* = *θ*, omitting the nonnegative terms, and taking into account the properties (10) and (12) yield

(34) $$\begin{array}{c} \frac{d}{2dt}{\left\|\varphi \right\|}^{2}+k_{1}{\left\|\varphi \right\|}_{V}^{2}\leq -\left(v\nabla \varphi ,\varphi \right)+a\left(\theta ,\varphi \right)\leq k_{1}{\left\|\varphi \right\|}_{V}^{2}+C_{3}\left({\left\|\varphi \right\|}^{2}+{\left\|\theta \right\|}^{2}\right), \end{array}$$

where *C*_3_ = ((1/4*k*_1_)‖**v**‖_*L*^∞^(*Q*)_^2^ + *a*/2);

(35) $$\begin{array}{c} \frac{d}{2dt}{\left\|\theta \right\|}^{2}\leq \kappa a\left(g\left(\varphi_{1}\right)-g\left(\varphi_{2}\right),\theta \right)\leq \kappa a\left\|\varphi \right\|\left\|\theta \right\|\leq \frac{\kappa a}{2}\left({\left\|\varphi \right\|}^{2}+{\left\|\theta \right\|}^{2}\right). \end{array}$$

Adding the last inequalities yields the estimate

(36) $$\begin{array}{c} \frac{d}{2dt}\left({\left\|\varphi \right\|}^{2}+{\left\|\theta \right\|}^{2}\right)\leq \left(C_{3}+\frac{\kappa a}{2}\right)\left({\left\|\varphi \right\|}^{2}+{\left\|\theta \right\|}^{2}\right). \end{array}$$

This estimate, along with the Gronwall inequality, implies that *φ* = *θ* = 0, which means the uniqueness of solutions.

## 5. Numerical Simulation

Numerical example involves a 2D square domain with the area of 3.24 mm^2^. It contains 64 holes corresponding to 32 inlets and 32 outlets that are interpreted as arteriolar and venular ends of the capillary network. The density of inlets and outlets is chosen in accordance with cerebral physiological characteristics reported in [16].

The following parameter values were used: *σ* = 0.03 (see [10]), *α* = 2.2 · 10^−3^mm^2^/s, *β* = 2.4 · 10^−3^mm^2^/s (see [7]), *a* = 39 s^−1^ (see [5, 17]), *b* = 9.2 mM (see [5]),  *ψ*_*H*_ = 3.6 · 10^−2^ mM (see [5]), *µ*_0_ = 0.08 mM/s (see [18]), *θ*_50_ = 5 · 10^−5^ mM (see [18]), and *r* = 2.73 (see [5]).

To specify the boundary conditions, we set *φ*_*b*_ = 9.2 mM, *θ*_*b*_ = 0.16 mM at the inlets and *φ*_*b*_ = 8.2 mM, *θ*_*b*_ = 0.076 mM at the outlets and at the edges of square. Moreover, we set *γ* = 1000*α*, *δ* = 1000*β*. The initial distributions of the concentrations are the following: *φ*_0_ = 4 mM and *θ*_0_ = 0.01 mM, which simulate the effect of tissue hypoxia.

**Figure 1:**
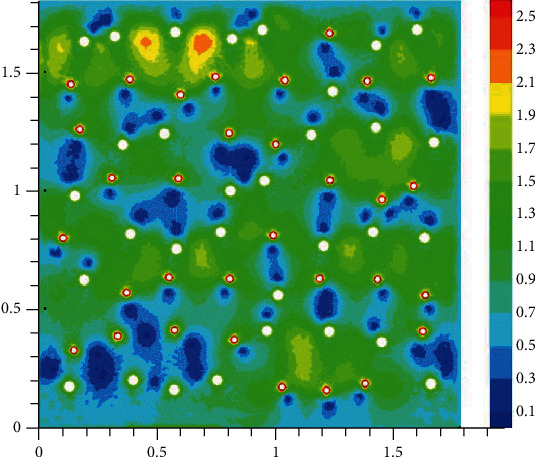
The absolute velocity (mm/s).

The velocity field **v** is computed in advance using the Stokes equation. To solve the Stokes equation, we set the following boundary conditions at the edges of the square: **v** = (0,0.6) mm/s. Moreover, following [19], velocities of 3.4 mm/s and 1.7 mm/s are set at the ends of arterioles and venules (boundaries of holes shown in Figure 1), respectively. Note that we use the Stokes equation to obtain an example of velocity field satisfying the specified boundary conditions in the considered perforated domain. Nevertheless (see Figure 1), in most of the domain, the velocity norm computed lies in the range of acceptable values (from 0.3 to 1.7 mm/s), which is necessary for normal functioning of brain cells (see [20]).

To approximate the initial-boundary value problem (1)–(5), the difference approximation of the time derivative and the first-order Finite Element spatial approximation were used. To resolve the nonlinearities at each time instant, the simple iterative procedure was applied. The function *g* (the inverse of *f*) was interpolated using cubic splines. It was observed that 20 steps of a simple iterative procedure provide a good accuracy in each time step. The Finite Element package FreeFEM++ (see [21]) was used to implement the solution method. To build a computational mesh, the following partition of the domain boundaries was specified: 60 segments for each edge of the square and 8 segments for each inlet and outlet. The time step length was taken to be equal to 0.25 s because of a good stability of the numerical scheme. Note that the Finite Element Method is suitable for solving the PDE models in complex geometries, for example, in the perforated domain considered here. Also, this approach is popular and developed enough for solving the diffusion-convection models. For example, for natural convection models in [22, 23], the stability and convergence analysis of the Finite Element Method is performed and its efficiency is demonstrated.

**Figure 2:**
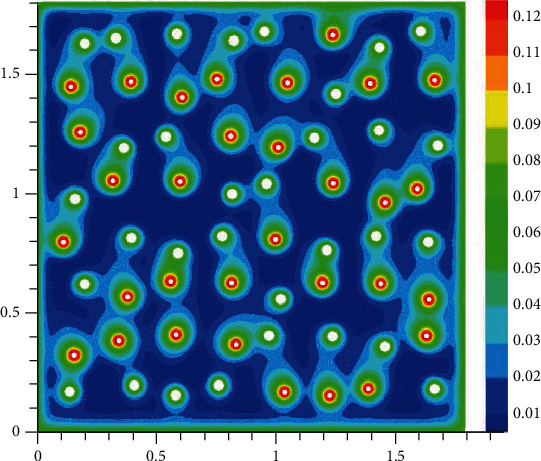
Brain tissue oxygen saturation after hypoxia: 1 second after oxygen begins to flow into the capillary network (mM).

**Figure 3:**
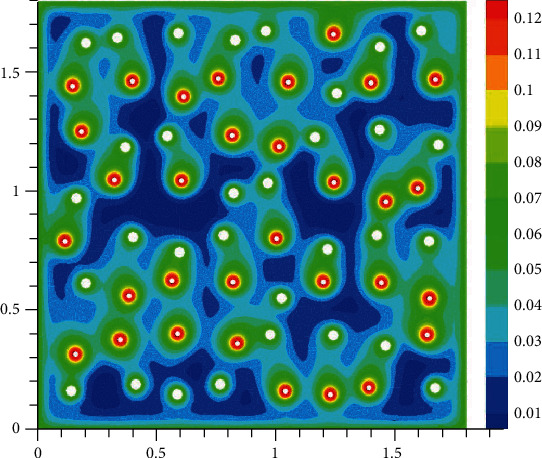
Brain tissue oxygen saturation after hypoxia: 2 seconds after oxygen begins to flow into the capillary network (mM).

**Figure 4:**
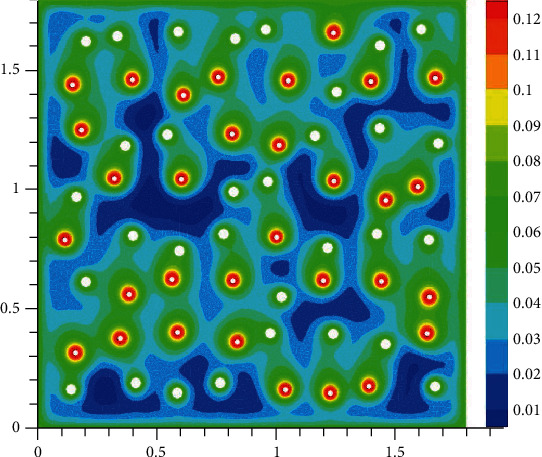
Brain tissue oxygen saturation after hypoxia: 3 seconds after oxygen begins to flow into the capillary network (mM).

**Figure 5:**
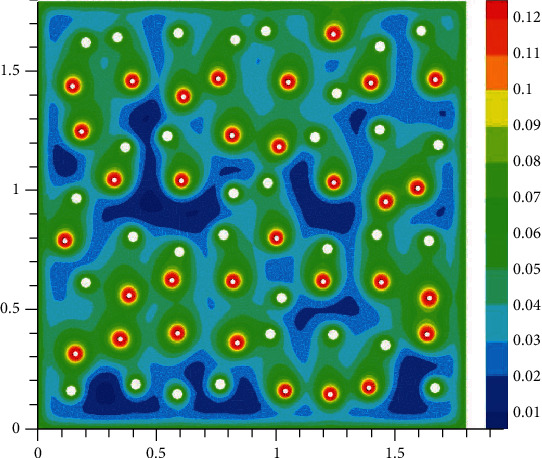
Brain tissue oxygen saturation after hypoxia: 7 seconds after oxygen begins to flow into the capillary network (mM).

The oxygen concentration in tissue at different time instants is shown in Figures 2–5. The dynamics of oxygen distribution is basically defined by the convection of oxygen in the blood fraction and its diffusion and consumption in tissue. Notice that a rapid stabilization (within 6-7 seconds) of oxygen distribution in tissue is observed. Nevertheless, more fast stabilization (within 3-4 seconds) occurs in the blood fraction.

## 6. Discussion

In the numerical example considered, the initial oxygen concentration in tissue corresponds to the effect of hypoxia, which is damaging for brain cells. Nevertheless, a quick supply of a sufficient amount of oxygen from the ends of arterioles into the homogenized capillary domain rapidly improves the situation so that the oxygen concentration is being stabilized at a safe level. This is in agreement with the estimation of time needed for oxygen to propagate in the capillary network and surrounding tissue. Indeed, assuming the number of inlets (ends of a1-arterioles) and outlets (ends of v1-venules) in an adult brain equals *N* = 76.8 · 10^6^, and the weight of the brain equals *m* = 1200 g (see [16]), and setting the density of the brain equals *ρ* = 1.04 g/cm^3^, we obtain a mean distance between inlets and outlets estimated by the value of $\sqrt[{3}]{m/\rho N}=0.25\text{ mm}$. Moreover, accounting for the speed of blood flow in the capillary network (see [20]), the time for which the blood flow passes from inlets to outlets is estimated by 2 seconds. Also, note that the maximal distance between brain cells and capillaries is in average of 0.02 mm (see [24]). With the diffusion coefficient of 0.0024 mm^2^/s (see [7]), oxygen molecules travel this distance in 0.1 second. Thus, in about 2.1 seconds after entering the capillary network, oxygen molecules begin to arrive the furthest parts of the brain. This estimation is in agreement with the results of our numerical experiment, which points out to the consistency of the continuum oxygen transport model considered.

A faster stabilization of oxygen concentration in the blood fraction compared with that in tissue is explained by the Hill equation (see [25]) determining the level of oxygen in plasma. The plasma oxygen level specifies the amount of oxygen penetrating from capillaries into tissue. According to the Hill equation, a small change in plasma oxygen level may lead to a relatively large change in tissue oxygen concentration if the oxygen concentration in blood is sufficiently large.

Thus, the considered continuum model of oxygen transport can be used to study the rate of oxygenation of brain tissue in dependence on the initial level of hypoxia as well as on blood oxygen concentrations at the inlets of capillary network (the ends of arterioles).

## Data Availability

The data used can be found in the references cited in this paper.
